# Cytokine removal: do not ban it, but learn in whom and when to use it

**DOI:** 10.1186/s13054-023-04736-8

**Published:** 2023-11-16

**Authors:** Bernhard M. W. Schmidt, H. Lang, Z. J. Tian, S. Becker, A. Melk

**Affiliations:** 1 Department of Nephrology and Hypertension, Hannover Medical School, Carl-Neuberg-Strasse 1, 30655 Hannover, Germany; 2 Department of Pediatric Kidney, Liver and Metabolic Diseases, Hannover Medical School, Carl-Neuberg-Strasse 1, 30655 Hannover, Germany

We are pleased that our publication has stimulated an important discussion as documented by the comment from Pappalardo et al. [1], who raised some issues regarding our manuscript “Efficacy of Cytosorb^®^-a systematic review and meta-analysis.”

The authors argue that mortality is not an adequate endpoint for interventions in intensive care medicine. Indeed, there might be endpoints, which are easier to reach, allow smaller sample sizes or shorter follow-up, and reflect some betterment in the clinical course. However, mortality is the most relevant endpoint and it is the ideal endpoint for a meta-analysis, as it is easy to measure and there is little variation in the way it is assessed. If a single intervention improves patient’s condition more than just providing easier handling, it should translate into a mortality benefit. This goal might be difficult to reach in a single study, but that is where strengths of meta-analyses come into play. By pooling the evidence, the results from small studies that each in itself have limited power can reach large impact.

We completely agree that treating the underlying disease and its relevant mediators is the most important task in intensive care medicine. That is where cytokine adsorption could indeed have its place. Harmful cytokine release can efficiently be treated, and doing this properly should then translate into a survival benefit. The problem is that we were not yet successful in defining the ideal situation and timing for the intervention. No evidence could be generated for positive effects of the device and, therefore, we do indeed believe that it should not be used widespread, but rather investigated further in well-designed RCTs for certain medical conditions. The commenters mention differing time periods during ECMO therapy [2], and we would suggest the first 24 (to 48) hours of sepsis. However, given the fact that there are also beneficial cytokines, there are definitely situations where we do not want protective factors to be removed [3].

We believe we have to decide what kind of medicine we want to practice: do we want to rely on personal experience, personal beliefs, or-at the best-logically sounding concepts and call it an “individualized approach” or do we want to rely on data. This means: do we want to base our medicine on eminence or evidence? We can only treat our patients individually, if proper data exist, which allows us to analyze the situation of each single patient in the context of therapeutic evidence. The fact that “patients are different” must not be used to whitewash therapy without evidence. Only proper data give us the knowledge and the justification to treat our patients individually.

In the case of Cytosorb^®^, this does not mean that we should not use it. Nevertheless, we have the obligation to perform RCTs in properly selected patients, taking into account the natural course of the respective disease in order to give us enough evidence for the safe usage of the device to the benefit of our patients.

## Data Availability

Not applicable.
